# Fine Mapping Identifies Candidate Genes Associated with Swine Inflammation and Necrosis Syndrome

**DOI:** 10.3390/vetsci12050508

**Published:** 2025-05-21

**Authors:** Katharina Gerhards, Sabrina Becker, Josef Kühling, Joel Mickan, Mirjam Lechner, Hermann Willems, Gerald Reiner

**Affiliations:** 1 Clinic for Swine—Herd Health Management and Molecular Diagnostics, Justus-Liebig-University Giessen, Frankfurter Strasse 112, 35392 Giessen, Germanygerald.reiner@vetmed.uni-giessen.de (G.R.); 2 UEG Hohenlohe, Am Wasen 20, 91567 Herrieden, Germany

**Keywords:** inflammation, tail lesions, animal welfare, swine

## Abstract

Swine inflammation and necrosis syndrome (SINS) is a widespread disease in pigs, causing pain, suffering, and damage. Inflammation is documented at different levels based on clinical signs, histopathology, clinical chemistry, metabolomics and transcriptomics. The influence of sow and boar, as well as a heritability of around 0.3, suggest a genetic component to the disease. The aim of the present study was to identify functional single nucleotide polymorphisms (SNPs) in the vicinity of gene markers previously mapped using GWAS. DNA samples were available from 234 already phenotyped piglets. These animals were re-sequenced with additional prior enrichment. The nine selected chromosomal regions cover a total length of 22 Mbp. The genome-wide association study (GWAS) revealed two series with a total of 15 significant missense polymorphisms on chromosomes 11, 14, and 15. The homozygous genotypes of the most discriminating SNPs in series 1 resulted in SINS scores of 3.5 and 17.9, respectively. Despite the partial linkage of the SNPs, interesting candidate genes were defined. The results allow a significant narrowing of the possible candidate genes for understanding the pathogenesis of SINS and for future use in selection breeding to overcome the syndrome. Further studies should be carried out on larger animal populations.

## Simple Summary

Swine inflammation and necrosis syndrome (SINS) is a widespread disease in pigs that causes pain, suffering, and damage, compromising animal health, performance, and consumer demand. In addition to husbandry, feeding, and management, genetics plays a crucial role in the development of and susceptibility to SINS. The aim of this study was to identify functional gene variants in the vicinity of known gene markers to better understand the development of the disease and to explore possibilities for targeted selection against SINS. To this end, DNA samples from 234 already phenotyped piglets were sequenced at a higher resolution. The study revealed 15 significant missense polymorphisms that have functional significance. The genotypes were associated with significant differences in the SINS phenotype (SINS score 3.5 vs. 17.9). These results provide the first evidence of functional genetic markers associated with SINS and open up the possibility of genetic selection against the disease.

## 1. Introduction

Improving animal welfare has become an important goal, especially for developed societies [1]. A newly defined but common disease in pigs with significant welfare implications is swine inflammation and necrosis syndrome (SINS) [2,3,4]. Symptoms begin with inflammatory loss of bristles, redness, and swelling. Later, exudative inflammation and even necrosis may occur [2]. Bleeding is also possible. The base of the tail, tip of the tail, ears, face, teats, navel, coronary bands, heels, and claws of newborn and suckling piglets, weaners, and finishers are affected. Macrophages and lymphocytes in the inflamed tails of piglets only a few hours old with intact epidermis show that SINS occurs without biting or technopathy, confirming the endogenous nature of the syndrome [5], although it can be modified by environmental conditions [2]. This endogenous component is also indicated by heritabilities around 0.3 for SINS traits [3]. Furthermore, significant and replicable breed and boar effects have been found [3,6]. SINS is widespread. Studies from Germany, France, and the Netherlands show that SINS affects over 60% of piglets on affected farms [2,3,4,7]. The associated inflammation and necrosis generally cause pain, suffering, and damage to the affected animals [8,9]. Improvements in housing conditions, in particular, the provision of hygienic drinking water from drinking troughs, the creation of opportunities for efficient thermoregulation, the reduction of protein and starch content in favor of crude fiber in feed, and the control of mycotoxins, can help control the syndrome [2,10], but are often difficult to implement in practice.

At the same time, detectable boar effects and moderate inheritance suggest the possibility of selecting against SINS and overcoming the syndrome [2,3]. Using boars that are less susceptible to SINS can result in higher birth and weaning weights and more piglets. At the same time, piglets experience less inflammation, necrosis, biting, and skin damage. This results in significantly improved animal welfare [3]. It is important to look for clues about the genes involved, not only for targeted selection based on functional markers, but also to better understand the pathogenesis of SINS. Studies of the liver transcriptome of piglets with varying degrees of SINS [11,12] allowed the clinical and pathohistological findings to be understood at the level of gene expression. Together with clinical chemistry and metabolomic results [11,13], they confirm a switch in the liver from normal anabolic metabolism to inflammatory and acute phase metabolism in SINS. A first genome-wide association study identified significant but not yet functional SNPs [14]. The significant SNPs associated with SINS traits were distributed across the entire genome with particularly high effects on SSC 7, 9, 11, 12, and 14–17. However, the relatively low coverage of this exploratory study has not yet revealed any functional SNPs.

The aim of the present study was to re-genotype selected regions of the piglets studied by Gerhards et al. [14] at a higher sequencing coverage to search for functional SNPs. Nine regions on eight chromosomes with the highest associations with SINS were selected.

## 2. Materials and Methods

### 2.1. Study Design

The study was based on the phenotypes and DNA of 234 piglets from the work of [14]. The phenotypes were used again because they were collected in a complex and precise manner, and no further experimental animals had to be used. The already published animal experiment was carried out in the conventional pig breeding stable of the Oberer Hardthof teaching and research station at Justus Liebig University Giessen under the approval of the authorities in Giessen, Germany with file number 708_M.

The environment was the same for all piglets studied. Sows and suckling piglets were housed together in 4.8 m^2^ farrowing pens with a plastic slatted floor. The sows were fixed in a farrowing crate. The floor for the sows was a cast iron slatted floor. Nipple drinkers and mother-child basin drinkers ensured the water supply for the animals. The lactating sows were fed according to their specific needs with a spotmix feeder in the trough.

The sow herd was a rotational cross of Topigs with German Landrace. The proportions of the individual genetics in the different sows were not the subject of detailed characterization. On the boar side, three purebred animals were selected that had been characterized for SINS on the basis of their progeny [6]. A Pietrain boar (PI) whose progeny had comparatively high SINS scores was designated high SINS PI (PI−). A second Pietrain boar whose progeny had comparatively low SINS scores was designated low SINS PI (PI+), and a Duroc boar whose progeny had the lowest SINS scores was designated as a low SINS Duroc boar (DU). The quantification of the SINS sensitivity of the boars results from [6]. There was no genetic relationship between these boars. The herd had a performance of 15 live born and 1.4 dead born piglets per sow.

The sows were artificially inseminated with the three boars that differed in terms of SINS. The boars were used in pairs as mixed semen, where the semen from two boars was mixed within one dose. This means that piglets from two different boars were present in each litter at the same time, with the high SINS Pietrain boar always acting as a positive control. This design was used to (i) limit the number of litters and experimental animals, (ii) to minimize environmental effects, (iii) to increase genetic variability within the piglets, (iv) to increase the number of sow–boar combinations, (v) to achieve a manageable number of piglets, and (vi) to minimize the variability of potential pathways involved in SINS sensitivity by using only three boars. At the same time, however, this small number of boars also led to stronger coupling of the SNPS.

The 27 sows produced 27 litters. In total, 234 anatomically normal piglets were used for further analysis. Their SINS phenotype was recorded at their third day of life. The father of the piglets was detected by paternity testing [14] (14 microsatellites in 2 multiplex PCRs, capillary gel electrophoresis) after phenotyping. This kind of order was chosen to ensure blinded data collection. The results of paternity testing revealed 14 mixed litters (from 14 sows) with 77 piglets from the PI+ and 39 piglets from the PI−, as well as 13 mixed litters from 13 sows with 48 piglets from the DU and 70 piglets from the PI−. The higher number of offspring from the PI− boar was due to the fact that it was always used as a control in the mixed semen experiment.

### 2.2. Clinical Scoring

The SINS phenotypes of the 234 piglets used in this study were taken from the previous study by [14].

In this study, inflammation and necrosis were recorded clinically, as already described in [5]. To ensure comparability with other studies, the piglets were scored on the day 3 of life. In all previous studies, clinical signs were clearly visible at this time, and the piglets were not yet as much exposed to environmental effects like weaners and fatteners. In order to minimize animal load, clinical signs were recorded using a digital camera (Canon EOS DC 8.1V, Canon, Krefeld, Germany) according to a standardized scheme for later detailed evaluation of the images (Windows Media Player, version 12, Microsoft GmbH, Munic, Germany).

Clinical alterations in the tail base and tail tip, the ears, the teats and navel, coronary bands, wall horn, heels, as well as the face were assessed individually. The following clinical characteristics were considered and scored 0 if the sign was not visible or 1 if the sign was visible (Table 1). The tail base and tail tip were independently scored for loss of bristles, swelling, redness, scab formation (tail tip only), rhagades, exudation, necrosis, bleeding (tail tip only), and ring-shaped constrictions (tail tip only). Ears were scored for a shiny skin, the loss of bristles, necrosis, and congested ear veins. The face was scored for the absence or presence of oedema at the eye lids and nose back. Teats were scored for swelling, reddening, scab formation, necrosis, and congested blood vessels. The navel was scored for signs of inflammation in the form of redness or swelling. Claws were scored qualitatively for any signs of inflammation at the coronary bands, walls, and heels. Scores for coronary bands and walls were averaged for front and hind limbs. Because the heels were drastically affected in some piglets, they were scored 0–3 for swelling and 0–3 for bleeding, each in front and hind limbs. The binary scores examined are presented by organ system as a percentage of affected piglets.

All findings were summed up to give an additive body part score (Table 1). This additive body part scores could range from 0 to 12 points. All of these body scores were summed in an unweighted manner to give the SINS score. This resulted in possible SINS scores between 0 and 44 for each piglet. All scores were assigned by two experienced persons using the four-eye principle. An overview of the evaluated phenotypes is given in Table 1. In addition, the SINS score after Z-transformation (ZSINS) was used.

### 2.3. DNA Extraction and Sequencing

DNA was extracted from docked piglet tails with the smart DNA prep (m) kit (Analytik Jena, Jena, Germany) and quantified using the Qubit dsDNA broad range assay kit (Invitrogen, Thermo Fisher Scientific, Waltham, MA, USA) on the Qubit Flex Fluorometer (Invitrogen, Thermo Fisher Scientific, Waltham, MA, USA). DNA was diluted to a uniform concentration of 50 ng/μL.

During shotgun library preparation, DNA (30–100 ng) was fragmented using the Fragmentase enzyme mix of the Allegro Targeted Genotyping Kit (TECAN/NuGEN, Männedorf, Switzerland). Indexed Illumina libraries were constructed using the Encore Rapid DR Multiplex System 1–96 (TECAN/NuGEN, Männedorf, Switzerland). All libraries were individually amplified for 8 cycles using MyTaq HS Red Mix (Meridian Bioscience, Bioline, Cincinnati, OH, USA) and standard Illumina primers in a 30 µL reaction volume. Additives were used to minimize PCR bias in library amplification (BioStab II PCR Optimizer, Sigma-Aldrich, St. Louis, MO, USA; Herculase II Fusion DNA Polymerase, Agilent, Santa Clara, CA, USA). The final libraries underwent quality control (QC) using agarose gel electrophoresis. If the concentration was slightly low, the libraries were run for up to 5 more PCR cycles. Up to 12 libraries were pooled for enrichment. Compiled pools were cleaned up using AMPure bead purification (using 0.8 Vol) followed by Monarch column purification (New England Biolabs, Ipswich, MA, USA).

Enrichment was performed using a ‘myBaits custom Enrichment kit’ (Daicel Arbor Biosciences, Ann Arbor, MI, USA), following the protocol for ‘most taxa’ and using 65 °C as the hybridization temperature. The only modification was the amplification of the enriched libraries. To avoid PCR bias, we again used the MyTaq HS Red Mix (Meridian Bioscience, Bioline, Cincinnati, OH, USA) with additives as described above. PCR reaction volumes for the enriched pool-DNAs were upscaled to a total volume of 200 µL. PCRs were run for 15 cycles.

Before sequencing, amplified library pools were purified with 0.8 Vol AMPure beads and size-selected on preparative agarose gel (350–650 bp fragments, 250–550 bp inserts).

The final QC of the DNA libraries was carried out using the Agilent Fragment Analyzer (Agilent, Santa Clara, CA, USA) and Qubit Fluorometer (Invitrogen, Thermo Fisher Scientific, Waltham, MA, USA).

Sequencing was performed on Illumina NextSeq 500 /NovaSeq 6000 (Illumina, San Diego, CA, USA). In this process, 150 bp paired-end reads with an average of 4 M read pairs per original sample were generated.

The *Sus scrofa* chromosomes (SSC) and regions selected for resequencing in this study were based on the SNPs with the highest probabilities of association with SINS traits identified in [14] (Table 2). These SNPs had effects of 8.1 and 8.4 (chromosome-wide) on SSC 9 and 12 and 8.9 to 16.8 (genome-wide) on SSC 7, 11, and 14 to 17. The region was defined around the corresponding SNP, and a range of 0.8 to 2.6 Mb was sequenced for each of these 9 regions.

### 2.4. OVarFlow Pipeline

For further analysis, the bz2 compressed fastq files received from the sequencing company were decompressed and converted to gz compressed fastq files.

The available raw data were transferred to the open-source workflow OVarFlow. OVarFlow is used for variant discovery of SNPs and insertion/deletion polymorphisms (Indels) in model and non-model organisms [15]. The workflow allows automation, documentation, and reproducibility of the individual analysis steps.

The reference genome and annotation Sscrofa11.1 (GCF_000003025.6; https://www.ncbi.nlm.nih.gov/datasets/taxonomy/9823/ (accessed on 19 May 2025)) were used for the implemented alignment, variant calling, and annotation. The min sequence length was set to 1.

The elements of the remaining analysis steps as well as their programs and versions used in the OVarFlow workflow correspond to those already described in the study by [14].

### 2.5. Genome-Wide Association Study (GWAS)

The preparation of the data output from OvarFlow for the GWAS and the actual execution of the GWAS was performed in R, version 4.2.1 [16]. RStudio [17] was used as the graphical user interface.

Since the enriched regions only extend to the autosomes, the gonosomes were not analyzed in the following. The annotated VCF file output at the end of the OVarFlow workflow was converted to binary PLINK format using the PLINK package version 1.9 [18,19].

The initial quality control of the genotype data was performed using PLINK. Only variants and individuals that passed these filter criteria were kept for the ongoing GWAS (missingness per marker: <0.01; missingness per individual: <0.1; minor allele frequency (MAF): >0.05; Hardy–Weinberg equilibrium (HWE): *p* > 0.000001).

A total of 63,362 variants were detected on the autosomes from 234 sequenced pigs.

A principal component analysis (PCA) was performed using TASSEL version 5.2 [20]. TASSEL 5.2 was also used to create a kinship matrix to account for kinship relationships from the offspring of the three different boars in further GWAS analysis.

The actual GWAS was conducted by GAPIT version 3 [21] using the BLINK model [22]. For this purpose, genotype data were converted from PLINK to hapmap format using TASSEL 5.2.

Boar effects, sow (litter), contemporary group, sex, birth weight, and PCA were used as fixed effects.

For PCA, four principal components were considered. Each sow was used only once in the study, so the effects of litter and sow were identical.

For the GWAS, Bonferroni-corrected significance thresholds were calculated genome wide (*p* ≤ 7.89 × 10^−7^) and chromosome wide (*p* ≤ 1.69 × 10^−4^–2.73 × 10^−5^) based on the 63,362 SNPs and a significance level of α = 0.05. All of the SNPs presented in the following overcome this significance threshold and can therefore be considered significant.

### 2.6. Variant Effect Prediction

For the functional annotation of the genetic variants found, information on the genetic coordinates, effect, and impact of each variant was provided. For this purpose, snpEff, version 5.0 [23] and Ensembl Variant Effect Predictor (VEP), release 113 [24] were used. In addition, the Sorting Intolerant From Tolerant (SIFT) score was given for non-synonymous mutations, which predicts whether an amino acid substitution could affect protein function based on sequence homology and the physical properties of the amino acids [25].

### 2.7. Statistical Analysis of SNP Effects on SINS Traits

Statistical analysis was performed using IBM SPSS version 29 (IBM, Munich, Germany). Body part scores and SINS scores were normally distributed. The analysis was performed using multivariate analysis of variance with the fixed effects of boar*piglet genotype, sex, and batch. The sow (litter) was used as a random effect in the model, and the birth weight of the piglets was used as a covariate. Interactions between effects were included. For the comparative estimation of the effects of boars and genotypes, a modified model was used instead of boar*genotype, with boar and genotype as fixed effects, while keeping all other effects the same. To characterize the boars, this model was modified by excluding the effect of the genotype. All SNPs were 100% linked in one of the two series. This linkage was cross chromosomal, i.e., it was not only haplotypes. This linkage is a consequence of the small number of boars. The genotypes of each SNP were designated as N (norm), and the deviating genotypes were designated as V (variant) according to the reference genome Sscrofa11.1. Due to the 100% agreement of the genotypes of all SNPs in a series, the individual SNPs were not evaluated further, but series 1 and 2, to which all SNPs were assigned, were evaluated. Means, standard errors (SE), and 95% confidence intervals (CI95) were presented. Prevalence was calculated by counting affected and unaffected individuals and presented in a purely descriptive manner. In ANOVA, differences were considered statistically significant at a significance level of α = 0.05. Since two series were tested according to Bonferroni, the lowest significance level was set at *p* ≤ 0.025. Some values that tended to differ were also shown in order not to lose any indication of possible effects.

The genotypes of the sow and boar were inferred from the genotypes of the piglets.

### 2.8. Annotation of Potential Candidate Genes

The NCBI Genome Data Viewer was used to search for positional candidate genes using the Sscrofa11.1 reference genome (https://www.ncbi.nlm.nih.gov/gdv/browser/genome/?id=GCF_000003025.6 (accessed on 19 May 2025)). A gene was considered to be a candidate gene if at least one significantly associated functional variant was located within the gene. Functional variants could include missense, splicing, and 5′ UTR variants.

Physiological functions of potential candidate genes were deduced using information from the NCBI database [26], GeneCards [27], PathCards [28], KEGG [29], MGI [30], and DAVID [31].

## 3. Results

### 3.1. Phenotypes

**Figure 1:**
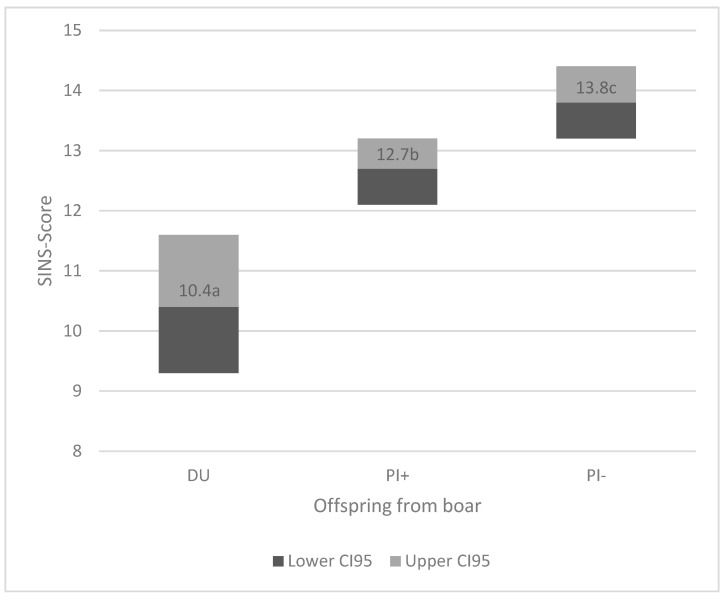
SINS scores in offspring from the three boars (DU = low SINS Duroc boar; PI+ = low SINS Pietrain boar, PI− = high SINS Pietrain boar). Number in box: mean; letter in box: groups with different letters were significantly different at *p* < 0.001; black box: lower 95% confidence interval (CI95) to mean; grey box: mean to upper 95% confidence interval.

The offspring of the three boars differed significantly in their SINS scores (*p* < 0.001) (Figure 1). The 95% confidence intervals for the offspring of DU, PI+, and PI− were between 9.3 and 11.6, 12.1 and 13.2, and 13.2 and 14.4, respectively.

In line with the significant differences in overall SINS scores, the offspring of the three boars also differed significantly in organ scores (except for claw wall). As expected, the offspring of the DU boar always had the lowest scores and those of the PI− boar always had the highest scores (Table 3). The greatest differences were found in the scores for tail base, tail tip, and teats, where the PI− offspring scores were about twice those of the DU offspring. The PI+ progeny scores were at the level of the DU progeny (tail base, tail tip, coronary bands), at the level of the PI− progeny (ears, heels), or in between (teats). With the exception of tail tip and teat alterations in Du offspring, the prevalence ranged from over 40 to over 90%. Again, Du offspring were always less affected, whereas PI− offspring had the highest prevalence in most cases.

### 3.2. Single Nucleotide Polymorphisms (SNPs)

The sequenced regions covered about 22 Mbp and revealed a total of 8668 SNPs that were significantly associated with at least one SINS phenotype. Twenty-two of these SNPs were functional and were used for further analysis (Table 4). Of these SNPs, 68.2% were missense variants (Missense) associated with an amino acid exchange in the protein; 22.7% were splice variants (Splice), where altered expression might be expected; and 9.1% were variants in the 5′ untranslated region (5′ UTR), i.e., in the regulatory region in or close to the promoter of a gene. The number of such SNPs on chromosomes 11, 14, and 15 was 10, 6, and 6, respectively. Due to the selected choice of the three boars, there was a strong association between the SNPs, which were grouped into one of two series. Series 1 was associated with SNPs on SSC11, SSC14, and SSC15. The second series was restricted to SSC15. The SNPs were located within or directly in the 5′ or 3′ UTR of defined genes. The SIFT scores of the resulting missense mutations were mostly classified as tolerated (SIFT score > 0.05) and in one case as deleterious (SNP 14_75945482; NUDT13 gene; SIFT score between 0.03–0.04).

The significance of series 1 SNP effects on the phenotypes, expressed as -LOG (*p*), was 7.5 (GWAS maximum for heels) and 19.2 (GWAS maximum for tail tip). The significance of the series 2 SNPs was 11.3 (GWAS maximum for tail base) (Table 4).

The genotypes of the piglets allowed a clear derivation of the genotypes of the sows and boars (Table 5). For the SNPs of series 1, the DU boar was homozygous for NN, and both Pietrain boars were homozygous for VV. The VV genotype predominated in the sows. The NN genotype only occurred in one sow. The distribution of genotypes in the piglets followed Mendel’s rules. The same picture was observed in series 2 (Table 5), although here the genotype NV was found in the PI− boar. Thus, the genotypes of the SNPs were largely assigned to the boars, and the diversity of the piglet genotypes resulted mainly from the genotypes of the sows.

**Figure 2:**
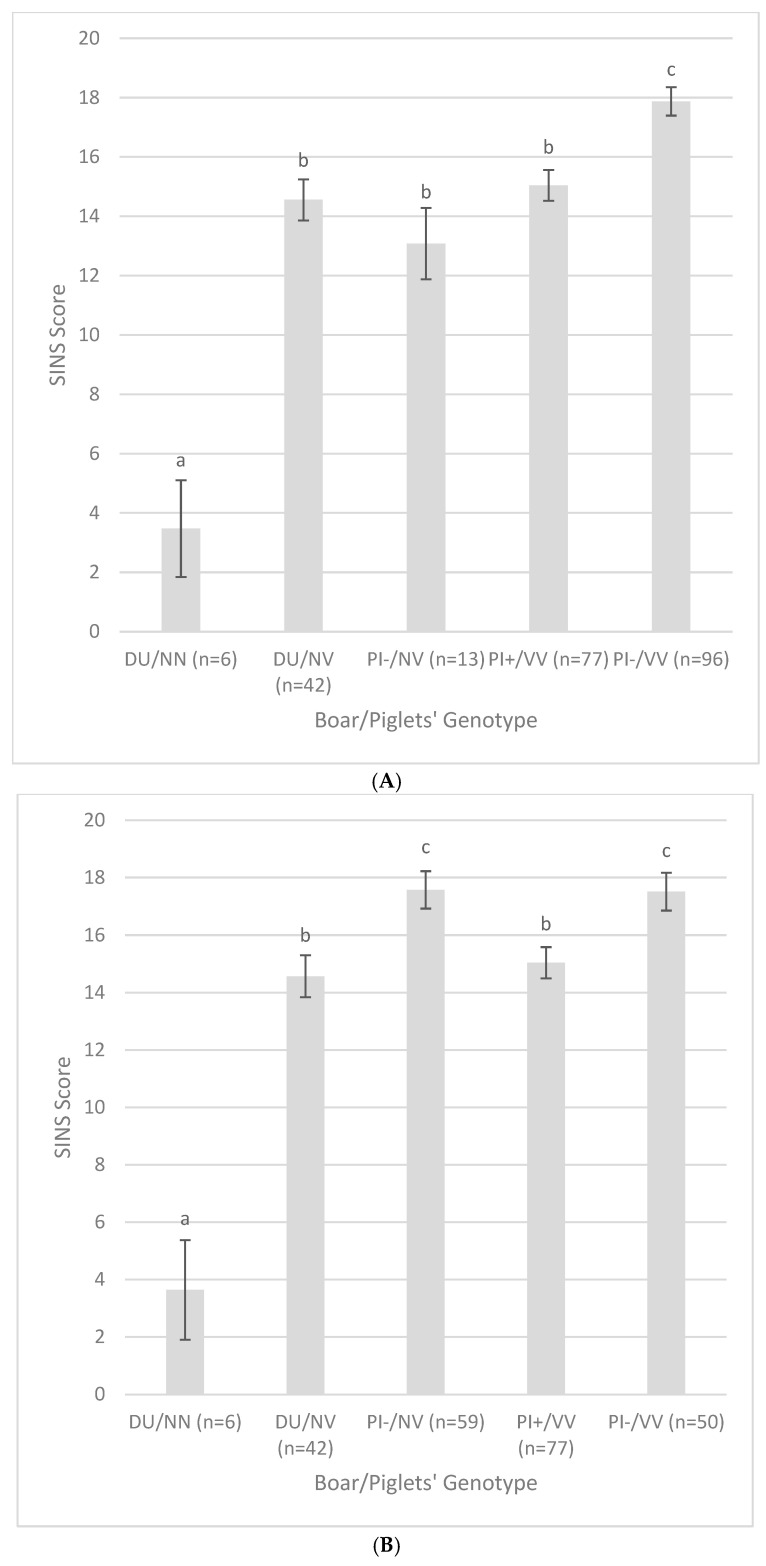
SINS scores by boar/piglet genotype combinations. (**A**): Series 1; (**B**): Series 2. DU = Low SINS Duroc boar; PI+ = Low SINS Pietrain boar, PI− = High SINS Pietrain boar; N: Norm allele as in the reference genome Sscrofa11.1; V: variant allele. Statistically significant differences are observed between columns with different letters (*p* < 0.05).

The favorable genotypes of all SNPs of both series occurred mainly in the progeny of the DU boar (Figure 2, Table 6). With regard to the SNPs of series 1 (Figure 2A, Table 6), the SINS values of the NN genotypes (mean = 3.5) were significantly lower than those of the NV genotypes (mean = 14.6) within the progeny of the DU boar and significantly lower than those of all other boar genotype combinations. At the same time, the scores of the PI− progeny with the VV genotype (mean = 17.9) were significantly higher than those of all other boar genotype combinations. In contrast, the SINS scores of the NV genotypes of the DU and PI boars and the VV genotypes of the PI+ boar did not differ. A similar picture emerged when considering series 2 (Figure 2B, Table 6), the only difference being that the PI− genotypes present were both associated with significantly higher SINS scores than all other boar–genotype combinations. For series 1, no difference could be observed between DU and PI− with respect to the heterozygous genotypes, whereas the VV genotypes differed significantly between PI+ and PI− progeny in favor of the PI+ piglets. In series 2, there was an additional significant disadvantage for the heterozygous genotype of the PI− offspring compared to the DU offspring. In both series, the increase from the NN to the NV genotype dominated, but this could only be reproduced in the DU offspring, as the NN genotype did not occur in the Pietrain offspring. This resulted in an overlap of boar effect and genotype, which could not be clearly separated due to the different distribution of genotypes in the boars. At the same time, however, the effect of increasing SINS scores from NN to NV to VV continued at comparable boar stages (DU, PI−).

The correlation for the SINS score was also largely reproduced at the level of individual body part scores, particularly for tail base, teats, coronary bands, and heels (Table 6).

**Figure 3:**
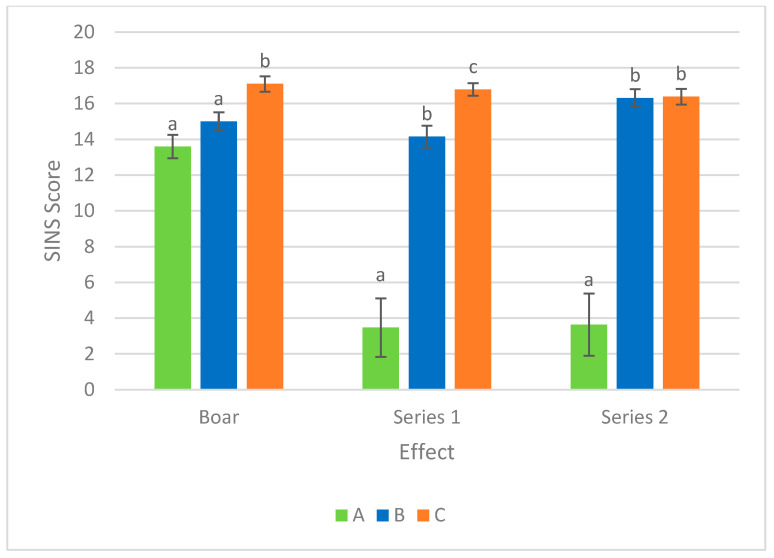
Effects of boar and SNP series on the SINS score. Boar: A = Low SINS Duroc; B = PI+ = Low SINS Pietrain; C = PI− = High SINS Pietrain; SNP series: A = NN; B = NV; C = VV; N: Allele as in the reference genome Sscrofa11.1; V: Variant allele. Statistically significant differences are observed between columns with different letters (*p* < 0.05).

When genotype and boar effect were simultaneously considered in the saturated model, the largest effect was found for series 1 and 2 (Figure 3). When the SINS scores of the progeny of the most favorable boar (DU) were 20% lower than those of the least favorable boar (PI−), the SINS scores of the NN genotypes were 79% lower than those of the VV genotypes in both series. The heterozygous genotypes were 16% (series 1) and 0% (series 2) below the values of the VV genotypes.

## 4. Discussion

Inflammation and necrosis of different body parts in pigs (SINS) often occurs at different age stages and affects the health and welfare of the animals [2,3,4,7]. In addition to husbandry and environmental influences [2,5,7], genetic influences on the expression of SINS have been demonstrated at the level of boar effects [6], breed effects [3,6], calculated heritabilities [3], and genomic associations [14] and confirmed using transcriptomic analyses [11,12]. The study by Kuehling et al. [6] showed significant differences between boars of the Duroc and Pietrain breeds in favor of the Duroc boars and between boars within the Pietrain breed. Therefore, a typical Duroc boar, whose progeny were hardly affected by SINS, and two Pietrain boars, whose progeny had the lowest (PI+) or highest (PI+) SINS score in the study by [6], were selected for the present study.

The piglets phenotyped and genotyped accordingly were already part of a genome-wide association study [14] with the aim of generating evidence of the genetic background of the extreme phenotypes. This study was closely accompanied by a transcriptome study [12]. By producing a number of offspring from these individual boars, it should in principle be possible to estimate the effects of specific SNPs. Building on the study by [14], 11 chromosomal regions with particularly high association to the SINS genotype were selected and sequenced with improved sequencing coverage compared to the original study. DNA and phenotypes were taken from the study by [14] in order to save experimental animals.

We deliberately selected only these three extreme boars for the study to reduce the possibility that different lines within a breed might use different pathways that interfere with each other and thus escape detection. However, this also means that there may well be alternative pathways for the regulation of SINS in other individuals and lines. More research is needed in this context. Of course, it was not the aim of the present study to identify differences between the breeds, and any information on differences between the progeny of the three boars can only refer to these three individual boars. Future studies on a larger number of boars are needed to make an overall statement at the breed level.

However, such studies can build directly on the results of the present study and thus save time and resources.

The pronounced chromosome-wide linkage of the SNPs in the present study is also a limitation of the chosen methodological approach. All identified functional SNPs were 100% linked in one of the two genotype series. Therefore, it is not possible to decide which of the SNPs in a series is actually functionally associated with the SINS phenotype. The reason for this is that when a boar is used, all SNPs of homozygous chromosome segments are only passed on to the offspring in a fully linked manner because crossing over has no effect. All linked SNPs will therefore show the same association with the phenotype. Future studies should therefore use several different boars to increase the variability of the SNPs adjacent to the functional SNP, thereby reducing haplotype blocks and breaking the linkage between adjacent SNPs. Nevertheless, in contrast to [14], the present approach identified the first functional SNPs associated with SINS and led for the first time to a manageable number of candidate genes that can be investigated in detail in the future. The association of any SNP in a series with the trait of interest must be seen as the gross effect of the 19 and 3 SNPs in series 1 and 2, respectively. It remains unclear how many of the SNPs are actually directly associated with the target traits and to what extent. The determination of the individual effects of the SNPs is reserved for future work, which would need to be carried out on larger numbers of boars and their progeny in order to separate the individual effects of the SNPs from each other. Future research with higher piglet and boar numbers could attempt to include animal models or haplotype-based GWAS to disentangle these effects.

It must further be taken into account that the phenotypes may be only partially explained by a certain SNP and that a residual variance of unexplained gene effects remains, which ultimately originates from unknown SNPs of the sow and boar.

The alleles of the SNP series were divided into N and V, with the N allele corresponding to the gene bank sequence (Sscrofa11.1) and the V allele to the variant deviating from it. The gene bank sequence originates from a pig of the Duroc breed. The basic assumption was that boars with extreme SINS scores should differ in the allele distribution of the SNPs associated with the phenotype. This hypothesis was confirmed. In relation to the animal species, there is also a clear association between the identified SNPs and the SINS score. However, the effects of the boars and the respective SNPs could not be completely separated. In a complete model, joining boar and SNP effects, however, the effects of the SNP series were estimated to be higher than the boar effects. The comparison of two genotypes (NN vs. NV in DU and NV vs. VV in PI−) also confirmed the directional relationship between SNPs and phenotype. However, it can be assumed that the respective boars should carry further favorable and unfavorable alleles in addition to the identified SNPs with regard to the SINS score, but these could not be detected due to the low effect sizes and the selected chromosome regions. Their identification would require very large numbers of animals.

The 19 SNPs in series 1 cover nine genes. Some of these genes meet the criteria for homologous physiological candidate genes for inflammation. Others show associations with performance parameters in swine or both. Negative side effects of selection for improved productivity that affect animal welfare have been widely reported [32,33]. The genes identified in the present study are interesting candidates for detecting such negative side effects. In addition, they may support the hypothesis that selection for performance may also have led to side effects in the form of inflammation and necrosis observed in SINS. Selection for performance can lead to favored gene variants and their associated selection along with alleles of other genes (selection signature/selective sweeps) [34,35,36]. Such genes can be physiologically unrelated to the performance trait, but involved in other traits [37], for example in the regulation of inflammation. On SSC11, three candidate genes are located within a known sweep region for growth, meatiness, and fertility in the pig.

One of the most interesting candidate genes of the present study is the protein lysine methyltransferase 21C (METTL21C), an important signaling molecule that modulates cellular plasticity in response to stress and ageing by modulating the NF-kB signaling pathway [38,39]. Previously known activities of METTL21C include heat shock protein binding [40,41] and response to oxidative damage during heat stress [38,42]. In addition, numerous studies demonstrate the role of METTL21C in myoblast formation, skeletal muscle development, and differentiation in poultry [39,43,44], which can lead to increased muscle weight and muscle fiber diameter. Steinert et al. [45] showed that the hypertrophic effect of METTL21C is partly due to the inhibition of protein degradation. By regulating the function of the uterus in laying hens, their production performance can be increased [46]. The gene is also associated with adaptability in chickens [47]. As poultry and pigs have undergone a very similar history of selection for conformation and muscle fiber thickness on the one hand, and for reproduction on the other, it is conceivable that a gene variant of METTL21C might have been selected for that has a negative effect on response to oxidative burst. The respiratory burst, which leads to the generation and accumulation of extracellular reactive oxygen species (ROS) to protect against invading pathogens, is one of the characteristic cellular processes observed during inflammation [48,49,50]. However, during oxidative stress, excessive ROS generation leads to imbalances that are associated with numerous pathologies, including cardiovascular disease [51,52,53,54]. Intracellular ROS has been shown to be involved in VEGF-dependent signaling in endothelial cells [55]. Thus, oxidative stress is directly linked to inflammation in a positive feedback loop [56]. Vasculitis plays a key role in the pathogenesis of SINS [5,10], and an intrinsic role of ROS, SOD, and VEGF has been demonstrated by transcriptomic studies [11,12,13]. In sheep and cattle, METTL21C has also been described in relation to stress signaling, oxidative metabolism, and calcium ion transport [57]. In the present study, a missense mutation and a mutation in the 5′ UTR of the SINS-associated METTL21C gene were found in series 1. There was a replacement of the small polar amino acid threonine by the non-polar amino acid methionine. When a missense mutation causes an amino acid change in a protein, it can have different effects depending on where it occurs in the protein and which amino acid is replaced by which other amino acid. It can lead to altered protein function, misfolding, and instability and be directly linked to disease; alternatively, it can be neutral and have no effect [58,59]. A base change in regulatory regions such as promoters or enhancers can affect the binding of transcription factors or other regulatory proteins. This can lead to a change in the expression of the gene, which in turn leads to a change in the amount of the responsible protein. In addition, SNPs in UTR regions can affect the stability and function of the mRNA and thus the amount of protein available [60]. Those substitutions that do not lead to a complete loss of protein function, but only to minor changes in the metabolic machinery, may be of particular interest in this context. For the coiled-coil domain containing 168 (CCDC168) gene, some partially deleterious SNPs were found in a selection scan region on SSC 11 when comparing wild boar and domestic pig sequences. This suggests that this gene is involved in a sweep mechanism, where the frequency of variants in a linked gene is strongly influenced as a result of selection for growth, meat or fertility [35]. It is discussed that CCDC168 may be associated with thermoregulatory ability in cattle and associated reduced fertility [61]. However, the functions of this gene are still largely unknown [62]. Based on the importance of thermoregulation for SINS [2] and the existing sweep effect, a candidate gene role for CCDC168 can also be considered. In the present study, six different missense mutations were identified. Among them were two substitutions of glutamic acid (acidic) by arginine (strongly basic). These substitutions are not considered deleterious, but may contribute to increased susceptibility to unfavorable thermoregulation, which plays a key role in the pathogenesis of SINS [2].

The basic immunoglobulin-like variable motif-containing protein (BIVM) gene has been associated with other selection criteria such as number of teats in Duroc pigs and improved reproduction [34,36]. In the present study, a splice variant was found.

SNPs that influence the splicing of the pre-mRNA by altering the splice site or by influencing the splicing regulators can lead to the formation of different protein variants, so-called isoforms, or to the loss of functional proteins [63]. It remains unclear how BIVM could be involved in the pathogenesis of SINS. However, the association of the gene variants with SINS susceptibility could be due to the fact that it may have arisen as a negative side-effect of selection for reproductive performance and number of teats.

ERCC5 (excision repair 5, endonuclease) is a paralog of BIVM and is involved in Nucleotide excision repair pathway [64]. ERCC5 is important for repairing DNA damage and has been linked to certain cancers and to the skin disease xeroderma pigmentosum [65].

NUDT13 (nudix-hydrolase 13) is involved in nucleotide metabolism. ECD (ecdysoneless cell cycle regulator) is involved in cell cycle regulation and has been detected in the peripheral blood transcriptome of pigs [66]. FAM149B1 (member of the family with sequence similarity 149, member B1) has been shown to be associated with renal function and dysfunction [67]. We did not find any homologous physiological overlap among ERCC5, NUDT13, ECD, FAM149B1, and SINS.

Regarding candidate genes from series 2, myosin IIIB (MYO3B) is associated with performance parameters (loin; fat ratio) in pigs [68,69]. In this context, it has been discussed that this gene may be responsible for the variation of other neighboring genes in the context of selection sweep [70]. In addition, associations with immune response were found [71]. Whether MYO3B has a direct or indirect effect on susceptibility to SINS could not be determined. None of the other candidate genes of series 2 appear to be directly related to inflammation and necrosis.

Current knowledge of the candidate genes and the pathways in which they are involved, particularly in pigs, does not yet allow a complete understanding of the genes in SINS pathogenesis. It is still unclear what effects the identified variants of the amino acid changes of the most interesting candidate genes might have on the phenotypes; however, for such key regulatory genes, we expect small functional differences rather than adverse effects on the target traits. Nevertheless, these results open up many possibilities for future research.

As the most common commercial boar breeds were used in the present study, it is likely that the identified candidate genes also play a role in other individuals of this breed. However, it is also possible that other pathways play an additional or even dominant role in other boars.

To answer this question, a larger study with significantly more boars of the same and other breeds and thus a greater variety of analyzed piglets is needed.

Based on the results of this and future studies, boars could be genotyped for the corresponding SNPs, and sires could be selected for breeding that carry as many favorable gene variants as possible. Therefore, these offspring would be less susceptible to SINS and can also pass this on to their offspring. Improving animal welfare has become an important goal, especially for developed societies [1], and breeding for increased performance has been identified as a major factor contributing to negative side effects on animal welfare [32,33]. This study provides evidence for candidate genes with gene variants that may be responsible for such negative side effects. These side effects occur in the form of SINS in a significant proportion of piglets on many farms, causing considerable pain, suffering, and damage [2]. Studies by several authors show that genetic selection for SINS is possible due to significant boar effects [6] and pronounced heritability [3], and that such selection would also result in significantly improved performance [3,4,10]. It could thus contribute to improved welfare through breeding [1,33,72,73]. It should be emphasized that this would not involve selecting for new gene variants that adapt animals to husbandry conditions, which would be critical in any case [72], but rather selecting against gene variants that have become widespread in populations due to breeding for increased performance and cause negative side effects. The candidate genes presented provide valuable approaches for confirmation in larger studies with a larger number of boars. Once favorable alleles are confirmed, they could be incorporated into practical genomic selection. Corresponding breeding values for boars could be recorded and economically weighted, and breeding targets could be adjusted to support husbandry, feeding, and management measures to combat SINS and improve animal welfare in pig production.

## 5. Conclusions

The present study demonstrates successful fine mapping by prior enrichment of certain chromosomal regions before sequencing. The analyzed regions were identified in a previous study using GWAS. This provided evidence for functional SNPs in some candidate genes that may contribute to the pathogenesis of SINS due to their homologous physiological function. The location of some of these SNPs in sweep regions may indicate that unfavorable gene variants for predisposition to SINS may have been favored by selection for performance in pigs. The results of this study can now be used as a starting point for further investigations on a larger number of piglets from a significantly larger number of boars. Ultimately, it is possible to assist in the fight against SINS by improving housing, feeding, and management conditions through practical genetic selection.

## Figures and Tables

**Table 1:** Overview of the collected individual characteristics and scores.

	Skin Shiny	No Bristles	Swelling	Red- ness	Scab Formation	Rhagades	Exudation	Necrosis	Bleeding	Ring- Shaped Constrictions	Vein Congestion	Edema		Additive Body Part Scores		Additive SINS- Score
Tail base	n.s.	0/1 ^3^	0/1	0/1	n.s.	0/1	0/1	0/1	n.s.	n.s.	n.s.	n.s.	→	0–6	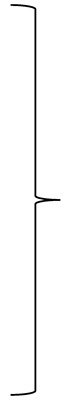	
Tail tip	n.s.	0/1	0/1	0/1	0/1	0/1	0/1	0/1	0/1	0/1	n.s.	n.s.	→	0–9		
Ears	0/1	0/1	n.s.	n.s.	n.s.	n.s.	n.s.	0/1	n.s.	n.s.	0/1	n.s.	→	0–4		
Face ^1^	n.s.	n.s.	n.s.	n.s.	n.s.	n.s.	n.s.	n.s.	n.s.	n.s.	n.s.	0/1	→	0–2		
Teats	n.s.	n.s.	0/1	0/1	0/1	n.s.	n.s.	0/1	n.s.	n.s.	0/1	n.s.	→	0–5		
Navel	n.s.	n.s.	0/1	0/1	n.s.	n.s.	n.s.	n.s.	n.s.	n.s.	n.s.	n.s.	→	0–2		0–44
Coronary bands ^2^	n.s.	n.s.	0/1	0/1	n.s.	n.s.	n.s.	n.s.	n.s.	n.s.	n.s.	n.s.	→	0–2		
Claw wall ^2^	n.s.	n.s.	0/1	n.s.	n.s.	n.s.	n.s.	n.s.	0/1	n.s.	n.s.	n.s.	→	0–2		
Heels ^2^	n.s.	n.s.	0–3	n.s.	n.s.	n.s.	n.s.	n.s.	0–3	n.s.	n.s.	n.s.	→	0–12		

n.s.: Not scored; ^1^: Eye lids and nose back are separately scored; ^2^: Hind and front limbs are separately scored; ^3^: Present (1) or absent (0).

**Table 2:** Chromosomes and regions selected for the present study based on the significance level of the major SNPs obtained by Gerhards et al. [14].

SSC ^1^	Start (Mbp)	End (Mbp)	Length (Mbp)	SNP Name	SNP Position	SNP Effect -log(*p*)
7	23.5	26.1	2.6	rs338509948	26025361	16.3
9	89.74	90.74	1	rs341512035	90241577	8.1
11	70.9	71.9	1	rs3475903338	71239679	8.9
12	43.6	44.4	0.8	rs1112423847	44738423	8.4
14	75.5	76.3	0.8	rs323488836	75918198	12.7
14	91.4	92.2	0.8	rs342612561	91808934	13.4
15	75	77	2	rs339270582	76926106	16.8
16	44	45	1	rs331217455	44669358	11.3
17	39.65	40.65	1	rs341628611	40157128	14.8

^1^ SSC: Sus scrofa chromosome; *p*: Significance.

**Table 3:** Mean, standard error, lower and upper 95% confidence intervals, and prevalence in offspring of the three boars (DU; PI+; PI−).

Score	Boar	Mean	SE	Lower CI95	Upper CI95	Prevalence (%)	*p*
Tail base	DU	0.77a ^1^	0.16	0.45	1.09	48.9	<0.001
	PI+	0.92a	0.13	0.67	1.17	52	
	PI−	1.45b	0.11	1.24	1.66	68.5	
Tail tip	DU	0.43a	0.15	0.13	0.73	29.8	0.012
	PI+	0.57a	0.12	0.34	0.81	45.3	
	PI−	0.92b	0.10	0.72	1.11	45.4	
Ears	DU	1.57a	0.15	1.28	1.87	78.7	<0.001
	PI+	2.59b	0.12	2.35	2.82	97.3	
	PI−	2.56b	0.10	2.36	2.75	97.2	
Teats	DU	0.74a	0.32	0.11	1.38	23.4	0.002
	PI+	1.59b	0.26	1.08	2.09	65.3	
	PI−	2.10b	0.21	1.68	2.52	60.2	
Coronary bands	DU	1.04a	0.13	0.78	1.31	61.7	<0.001
	PI+	0.89a	0.11	0.68	1.10	61.3	
	PI−	1.40b	0.09	1.22	1.57	83.3	
Claw wall	DU	1.60	0.09	1.42	1.78	83	n.s.
	PI+	1.81	0.07	1.67	1.96	96	
	PI−	1.72	0.06	1.60	1.84	89.8	
Heels	DU	5.91a	0.28	5.36	6.47	93.6	(0.055)
	PI+	5.75ab	0.22	5.31	6.18	100	
	PI−	6.42b	0.19	6.05	6.78	99.1	

SE: Standard error; *p*: Significance; DU = Low SINS Duroc boar, PI+ = Low SINS Pietrain boar, PI− = High SINS Pietrain boar; ^1^: Values within one phenotype with different letters are statistically significant at *p* < 0.05.

**Table 4:** Associated functional SNPs by series, position, and phenotype with significance, base exchange, and meaning.

Series ^1^	SSC ^2^	Position	Phenotype 1 ^3^		Phenotype 2 ^3^		Base		Gene	Triplet		AA		Direction ^6^	Ex-on	Effect ^7^	Meaning (SNPEff)
			trait	-LOG(*p*)	trait	-LOG(*p*)	N ^4^	V ^5^		N ^4^	V ^5^	N ^4^	V ^5^				
1	11	71015446	Heels	7.5	Tail tip	19.2	G	A	METTL21C	CGT	CAT	T	M	r	1	Missense	MOD
1	11	71020888	Heels	7.5	Tail tip	19.2	T	C	METTL21C						1	5′UTR	LOW
1	11	71054195	Heels	7.5	Tail tip	19.2	T	C	CCDC168	CTG	CCG	E	R	r	1	Missense	MOD
1	11	71054252	Heels	7.5	Tail tip	19.2	T	C	CCDC168	ATT	ACT	N	S	r	1	Missense	MOD
1	11	71056181	Heels	7.5	Tail tip	19.2	C	T	CCDC168	ACG	ATG	R	H	r	1	Missense	MOD
1	11	71056239	Heels	7.5	Tail tip	19.2	T	C	CCDC168	CCT	CCC	R	G	r	1	Missense	MOD
1	11	71056968	Heels	7.5	Tail tip	19.2	C	G	CCDC168	CTC	CTG	Q	E	r	1	Missense	MOD
1	11	71057564	Heels	7.5	Tail tip	19.2	T	C	CCDC168	CTG	CCG	E	R	r	1	Missense	MOD
1	11	71114837	Heels	7.5	Tail tip	19.2	A	G	BIVM						2	Splice	LOW
1	11	71167929	Heels	7.5	Tail tip	19.2	G	A	ERCC5	CGT	CAT	R	H	f	15	Missense	MOD
1	14	75945482	Heels	7.5	Tail tip	19.2	G	C	NUDT13	AGA	ACA	R	T	f	2	Missense	MOD
1	14	75953186	Heels	7.5	Tail tip	19.2	A	G	NUDT13	ATG	GTG	M	V	f	4	Missense	MOD
1	14	75956113	Heels	7.5	Tail tip	19.2	G	C	NUDT13	CGA	CCA	R	*p*	f	7	Missense	MOD
1	14	75977767	Heels	7.5	Tail tip	19.2	C	T	ECD						5	Splice	LOW
1	14	75998607	Heels	7.5	Tail tip	19.2	C	T	FAM149B1	ACA	ATA	T	I	f	3	Missense	MOD
1	14	76053610	Heels	7.5	Tail tip	19.2	A	G	FAM149B1	AAG	AGG	K	R	f	11	Missense	MOD
1	15	75617207	Tail tip	19.2	Heels	7.5	T	C	LRP2	GAT	GAC	I	V	r	33	Missense	MOD
1	15	75626516	Tail tip	19.2	Heels	7.5	A	C	LRP2						41	Splice	LOW
1	15	76939828	Tail tip	19.2	Heels	7.5	G	T	ERICH2	promotor					1	5′UTR	LOW
2	15	75405294	Tail base	8.4			G	T	ABCB11						2	Splice	LOW
2	15	76734995	Tail base	8.4			A	G	MYO3B	AGT	GGT	S	G	f	30	Missense	MOD
2	15	76646899	Tail base	11.3			A	G	MYO3B						22	Splice	LOW

^1^: All SNPs were linked in two series; ^2^: Sus scrofa chromosome; ^3^: Up to two different phenotypes were significantly associated in the GWAS (see -log(*p*)); ^4^: N = Norm relative to the reference genome Sscrofa11.1; ^5^: V = Variant deviating from the reference genome Sscrofa11.1; ^6^: direction: f = 5′ to 3′; r = 3′ to 5′; ^7^: Splice = Splicing region variant, 5′UTR = 5′ Untranslated region variant, Missense = Missense variant.

**Table 5:** Sow and boar genotypes derived from piglet genotypes.

	Boar		Sow		Piglets	
Series	Breed	Genotype	Genotype	n	Genotype	n
1	DU	NN	VV	12	NV	42
			NN	1	NN	6
	PI+	VV	VV	14	VV	77
	PI−	VV	VV	21	VV	96
			NV	5	NV	13
2	DU	NN	VV	12	NV	42
			NN	1	NN	6
	PI+	VV	VV	14	VV	77
	PI−	NV	VV	26	VV	59
					NV	50

**Table 6:** SINS scores of the individual body parts by boar/piglet genotype combination and series.

	DU/NN	DU/NV	PI−/NV	PI+/VV	PI−/VV	*p*
Series 1						
Tail base	0.15 ± 0.37 a	0.73 ± 0.15 a	1.11 ± 0.27 b	0.88 ± 0.12 a	1.5 ± 0.11 b	<0.001
Tail tip	0.44 ± 0.41	0.56 ± 0.17	0.35 ± 0.30	0.65 ± 0.13	0.77 ± 0.12	n.s.
Ears	−0.13 ± 0.30 a	1.7 ± 0.13 b	1.2 ± 0.22 c	2.51 ± 0.1 d	2.80 ± 0.09 d	<0.001
Teats	0.1 ± 0.72 a	1.05 ± 0.3 ab	1.1 ± 0.53 ab	1.62 ± 0.23 b	2.32 ± 0.21 c	<0.001
Coronary bands	−0.11 ± 0.34 a	1.15 ± 0.14 bc	1.4 ± 0.25 bc	0.94 ± 0.11 b	1.36 ± 0.10 c	<0.001
Claw wall	0.15 ± 0.17 a	1.71 ± 0.07 b	1.86 ± 0.13 b	1.82 ± 0.05 b	1.71 ± 0.05 b	<0.001
Heels	1.93 ± 0.7 a	6.47 ± 0.29 c	4.94 ± 0.51 b	5.61 ± 0.22 b	6.44 ± 0.20 c	<0.001
						
Series 2						
	DU/NN	DU/NV	PI+/VV	PI−/NV	PI−/VV	*p*
Tail base	0.1 ± 0.4 a	0.7 ± 0.2 a	0.9 ± 0.1 a	1.8 ± 0.1 b	1.3 ± 0.1 c	<0.001
Tail tip	0.5 ± 0.4	0.6 ± 0.2	0.6 ± 0.1	0.9 ± 0.1	0.7 ± 0.1	n.s.
Ears	−0.1 ± 0.3 a	1.7 ± 0.1 b	2.5 ± 0.1 c	2.7 ± 0.1 c	2.7 ± 0.1 c	<0.001
Teats	0.1 ± 0.8 a	1 ± 0.3 ac	1.6 ± 0.2 bc	2.1 ± 0.3 b	2.1 ± 0.3 b	0.018
Coronary bands	0 ± 0.3 a	1.2 ± 0.1 bc	0.9 ± 0.1 b	1.3 ± 0.1 c	1.4 ± 0.1 c	<0.001
Claw wall	0.1 ± 0.2 a	1.7 ± 0.1 b	1.8 ± 0.1 b	1.7 ± 0.1 b	1.8 ± 0.1 b	<0.001
Heels	2.1 ± 0.7 a	6.5 ± 0.3 b	5.6 ± 0.2 c	6.1 ± 0.3 b	6.7 ± 0.3 b	<0.001

DU = Low SINS Duroc boar; PI+ = Low SINS Pietrain boar, PI− = High SINS Pietrain boar; N: Allele as in the reference genome Sscrofa11.1, V: Variant allele; *p*: Significance. Statistically significant differences are observed between values in rows with different letters (*p* < 0.05).

## Data Availability

All data are available from the corresponding author by reasonable request.

## Abbreviations

The following abbreviations are used in this manuscript:

ABCB11	ATP-binding cassette, subfamily B member 11
BIVM	Basic immunoglobulin-like variable motif-containing protein
CCDC168	Coiled-coil domain containing 168
CI95	95% confidence interval
DU	Low SINS Duroc boar
ECD	Ecdysoneless cell cycle regulator
ERCC5	Excision repair 5, endonuclease
ERICH2	Glutamate rich 2
f	Forward (5′–3′)
FAM149B1	Member of the family with sequence similarity 149, member B1
GWAS	Genome-wide association study
HWE	Hardy–Weinberg equilibrium
Indels	Insertion/deletion polymorphisms
LRP2	LDL receptor related protein 2
MAF	Minor allele frequency
METTL21C	Protein lysine methyltransferase 21C
Missense	Missense variant
MOD	Modifier
MYO3B	Myosin IIIB
N	Norm (e.g., base, triplet, amino acid (AA)) according to reference genome Sscrofa11.1
NN	Homozygous genotype of the norm variant
NUDT13	Nudix-hydrolase 13
NV	Heterozygous genotype
n.s.	Not significant
P	Significance
PCA	Principal component analysis
PI	Pietrain boar(s)
PI+	Low SINS Pietrain boar
PI−	High SINS Pietrain boar
QC	Quality control
r	Reverse (3′–5′)
SE	Standard error
SIFT	Sorting Intolerant From Tolerant Score
SINS	Swine inflammation and necrosis syndrome
SNPs	Single nucleotide polymorphisms
Splice	Splicing region
SSC	Sus scrofa chromosome
UTR	Untranslated region
V	Variant (e.g., base, triplet, amino acid (AA)) deviating from the reference genome Sscrofa11.1
VEP	Variant Effect Predictor (Ensembl)
VV	Homozygous genotype of the variant
ZSINS	SINS score after Z-transformation
